# How do bacterial endosymbionts work with so few genes?

**DOI:** 10.1371/journal.pbio.3002577

**Published:** 2024-04-16

**Authors:** John P. McCutcheon, Arkadiy I. Garber, Noah Spencer, Jessica M. Warren

**Affiliations:** 1 Biodesign Institute and School of Life Sciences, Arizona State University, Tempe, Arizona, United States of America; 2 Howard Hughes Medical Institute, Chevy Chase, Maryland, United States of America; University of Oxford, UNITED KINGDOM

## Abstract

The move from a free-living environment to a long-term residence inside a host eukaryotic cell has profound effects on bacterial function. While endosymbioses are found in many eukaryotes, from protists to plants to animals, the bacteria that form these host-beneficial relationships are even more diverse. Endosymbiont genomes can become radically smaller than their free-living relatives, and their few remaining genes show extreme compositional biases. The details of how these reduced and divergent gene sets work, and how they interact with their host cell, remain mysterious. This Unsolved Mystery reviews how genome reduction alters endosymbiont biology and highlights a “tipping point” where the loss of the ability to build a cell envelope coincides with a marked erosion of translation-related genes.

## Abstract 2

The move from a free-living environment to a long-term residence inside a host eukaryotic cell has profound effects on bacterial function. This Unsolved Mystery article explores how genome reduction alters endosymbiont biology and highlights a ‘tipping point’ where the loss of the ability to build a cell envelope coincides with a marked erosion of translation-related genes.

## Introduction

Host-beneficial endosymbiosis, where an organism stably maintains an unrelated organism inside some or all of its cells, is a complex process. The host must calm its immune system sufficiently to not kill its endosymbiont or overreact to the sustained presence of a foreign cell. The host must extract what it needs—often nutrition, energy, or protective molecules—from the endosymbiont without taking so much that it imperils the health of its smaller partner. How the endosymbiont experiences host cell restriction is difficult to assess, but genome reduction, gene loss, and rapid rates of sequence evolution are near-universal outcomes of the process. The endosymbionts with the smallest genomes have lost so many genes that they lack the ability to do much at all on their own and start to resemble mitochondria and chloroplasts more than typical bacteria. How these hosts and endosymbionts integrate their biochemical and cell biological processes is largely unknown.

In this Unsolved Mystery, we briefly outline which bacteria become endosymbionts, which genes are retained in endosymbionts, and what happens to the genome and protein composition of endosymbionts. We focus on endosymbionts with genomes less than 200 kilobases (kb) in length because these organisms seem to have crossed a tipping point where gene loss has been so extensive that it is unclear how fundamental bacterial processes are carried out. We then highlight 4 unsolved mysteries related to the function of endosymbionts with highly reduced genomes: how to build a cell boundary with no genes to make it; how to transport molecules across the cell envelope with no genes to make transporters; how to make proteins when missing key translation-related genes; and how proteins function with extreme amino acid compositional biases.

## Features of endosymbionts with reduced genomes

### Symbionts with highly reduced genomes come from across the bacterial tree of life

**Fig 1:**
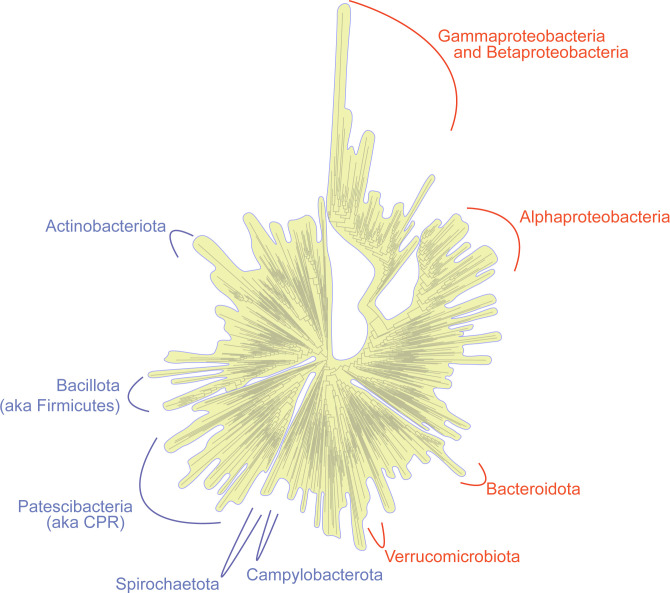
Small genome endosymbionts are derived from diverse bacteria. A phylogenetic tree of bacteria, where major groups containing organisms with genomes of less than 1 Mb are noted in blue, and groups with organisms containing genomes less than 200 kb are shown in red. The curved lines represent the approximate position of these groups on the tree. The yellow outline on the tree is there to de-emphasize the precision of the branches and to remind the reader that the group locations are approximate. The tree structure and phyla location were adapted from [1].

Most free-living bacteria have genome sizes greater than 1 megabase (Mb) in length. Bacteria with genomes less than 1 Mb are almost exclusively bacteria that live in (endosymbionts) or on (ectosymbionts) other organisms. Fig 1 shows a tree representing all known bacterial diversity [1,2], with major high-level bacterial groups (that is, phyla) containing representatives with <1 Mb genomes indicated by name. The point is not the exact names, which are in flux [2], but rather to show that many different bacterial groups, from all over the bacterial tree, have become symbionts with reduced genomes.

It is difficult to comprehend the antiquity and diversity represented in Fig 1. Bacteria are extremely old and extraordinarily diverse. For example, even the familiar (and nearly identical-looking) *Escherichia* and *Salmonella*, which are so similar that they are not differentiable on the tree in Fig 1, are estimated to have diverged approximately 120 million years ago [3], roughly the time of modern birds’ divergence from dinosaurs [4]. There are 10 bacterial groups containing representatives with genomes <1 Mb (Fig 1 in blue and red). The bacteria that have the most severely reduced genomes, which we define here as genomes less than 200 kb (or about 200 genes), are restricted to 5 bacterial groups (Fig 1 in red). While 5 phyla might seem like a somewhat limited amount of bacterial diversity, the Alphaproteobacteria, Betaproteobacteria, and Gammaproteobacteria are estimated to have shared a common ancestor about 2.5 billion years ago [5]. Adding the Bacteroidia and Verrucomicrobiota pushes back the common ancestor of these bacterial groups to near the origin of cellular life >3 billion years ago [5]. The shared genomic features we highlight in the next section are therefore notable and point to convergent evolutionary paths towards endosymbiosis.

### Some endosymbionts retain very few genes

The extreme level of genome reduction experienced by endosymbionts has been reviewed elsewhere [6–9]. In this Unsolved Mystery, we ignore endosymbiont genes related to host function, such as those for provisioning nutrients, protective molecules, or energy. Instead, we focus on patterns of gene retention in 2 core functional categories: genes to build (and transport across) a cell envelope and genes involved in translating mRNA into protein.

**Fig 2:**
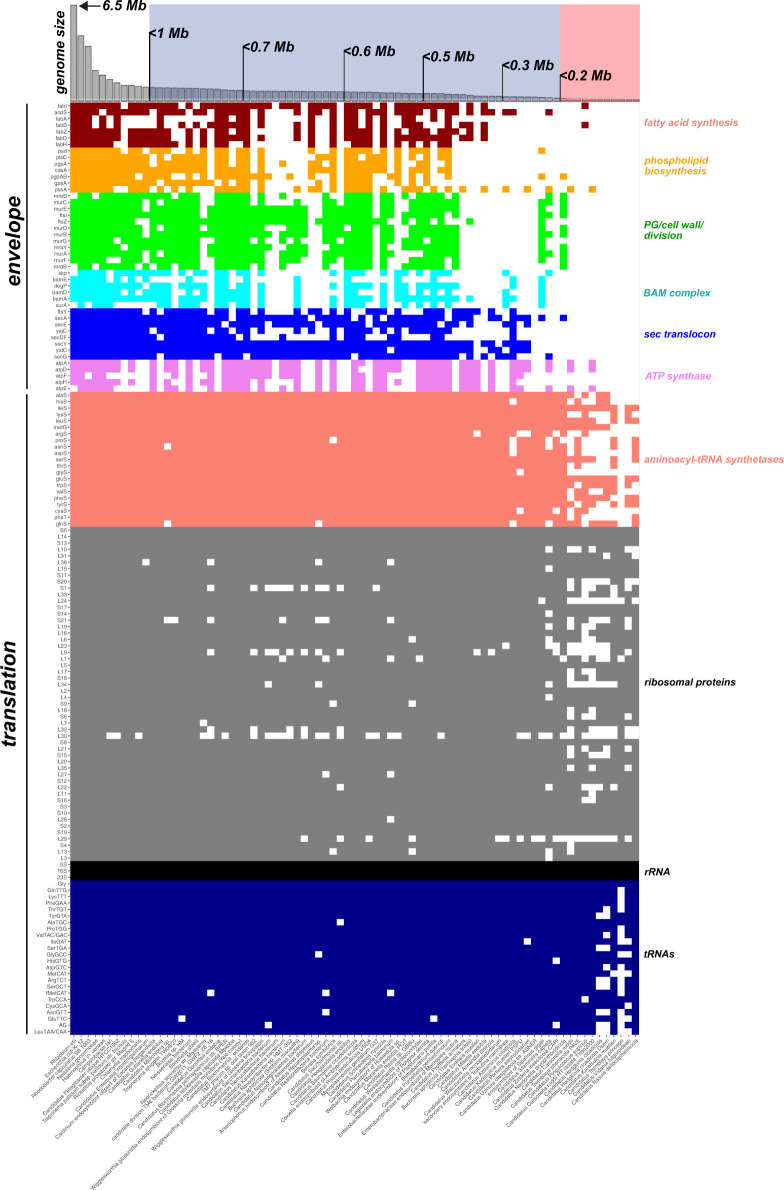
Loss and retention of cell envelope and translation-related genes in bacteria with reduced genomes. The sizes of representative genomes of less than 1 Mb, with a few larger genomes included, are arrayed across the top as grey bars in decreasing genome size from left to right. Bars boxed in blue are less than 1 Mb but greater than 200 kb, those boxed in red are the tipping-point endosymbionts with genomes less than 200 kb. Envelope-related genes are separated by categories where a colored box indicates the gene is present and a white box indicates a gene is absent. Translation-related genes are similarly arrayed on the bottom part of the figure. In general, the complete loss of the ability to autonomously make a cell envelope (fatty acids, phospholipids, cell wall) occurs in bacteria with genomes smaller than about 400 kb, and these losses coincide with losses in the ability to transport macromolecules across (BAM complex, sec translocon) or insert into (ATP synthase) lipid bilayers. Genomes less than 200 kb start to lose a significant number of ribosomal proteins, tRNAs, and amino acyl-tRNA synthetases. Organisms for this figure were chosen by manually selecting species from the GenBank prokaryotes list. All organisms with genomes less than 1 Mb were included except in cases where multiple examples from the same genus were present, where the largest and smallest genome from the genus were selected. Genomic data for these organisms was downloaded from GenBank using the bit software toolkit [11]. Gene presence and absence were calculated from a combination of literature review, existing GenBank annotations, and by performing searches against HMMER [12] profiles in the Pfam and TIGRFAM databases. We caution that some genomes included are in draft form, and so the exact gene patterns should be considered tentative. The code and raw data used to generate this figure are available at https://zenodo.org/records/10780716.

Fig 2 shows a gene loss and retention matrix for a representative set of bacteria with genomes of less than 1 Mb. Two main patterns emerge. The first is that genes for building cell envelopes (fatty acids, phospholipids, cell wall, etc.), transporting proteins across these envelopes (BAM complex, sec translocon), and proteins that function at the envelope (ATP synthase) are generally lost in concert with each other. This makes sense, because if a bacterium cannot build an envelope on its own, it becomes difficult to control transport across and insert transmembrane proteins into its membranes. It is notable that severe erosion in these pathways can occur in bacteria with somewhat, but not extremely, reduced genomes (on the order of 0.6 to 0.7 Mb), with many of these examples being candidate phyla radiation bacteria (also known as Patescibacteria) [10]. But as endosymbionts experience genome reduction beyond about 400 kb, they almost universally lose the autonomous ability to build and to control the transport of molecules across their cellular envelope.

The second pattern, which has been described many times before [7,8,13], is that certain key genes related to information processing—genome replication, transcription, and translation—are tightly retained by all bacteria, even those with severely reduced genomes. What we highlight here is that there is a genome size tipping point at approximately 200 kb where even the most tightly conserved process—translation—starts to erode (Fig 2). Genomes above this 200 kb size boundary mostly retain small sets of DNA replication and RNA transcription genes [8], enough tRNA genes to decode all codons, enough aminoacyl-tRNA synthetase (aaRS) genes to charge all of their tRNAs with amino acids, and a near-complete set of about 50 ribosomal protein genes. Genomes below this 200 kb threshold have lost many aaRS, ribosomal protein, and tRNA genes (Fig 2). Hereafter, we will refer to endosymbionts with genomes less than 200 kb as tipping-point endosymbionts.

### Endosymbionts have extraordinarily biased genomes and proteomes

Bacterial genomes show large differences in GC and AT base pair frequencies, ranging from approximately 75% GC (approximately 25% AT) to approximately 13% GC (approximately 87% AT) [14]. While the forces that drive these GC content differences remain enigmatic [15], there is a strong link between the GC content of a genome and the frequencies of amino acids encoded by that genome [16,17]. This link is due to the near-universal nature of the genetic code and because codon sets for some amino acids are more GC or AT rich than others. For example, proteomes encoded by very GC-rich genomes will tend to be alanine rich (encoded by GCA, GCT, GCC, and GCG codons), and proteomes encoded by very AT-rich genomes will tend to be lysine rich (encoded by AAA and AAG codons). Endosymbiont (and organelle) genomes are often very AT rich [18,19], and this genomic AT bias affects the amino acid composition of some endosymbiont [20] and organelle [21] proteomes.

**Fig 3:**
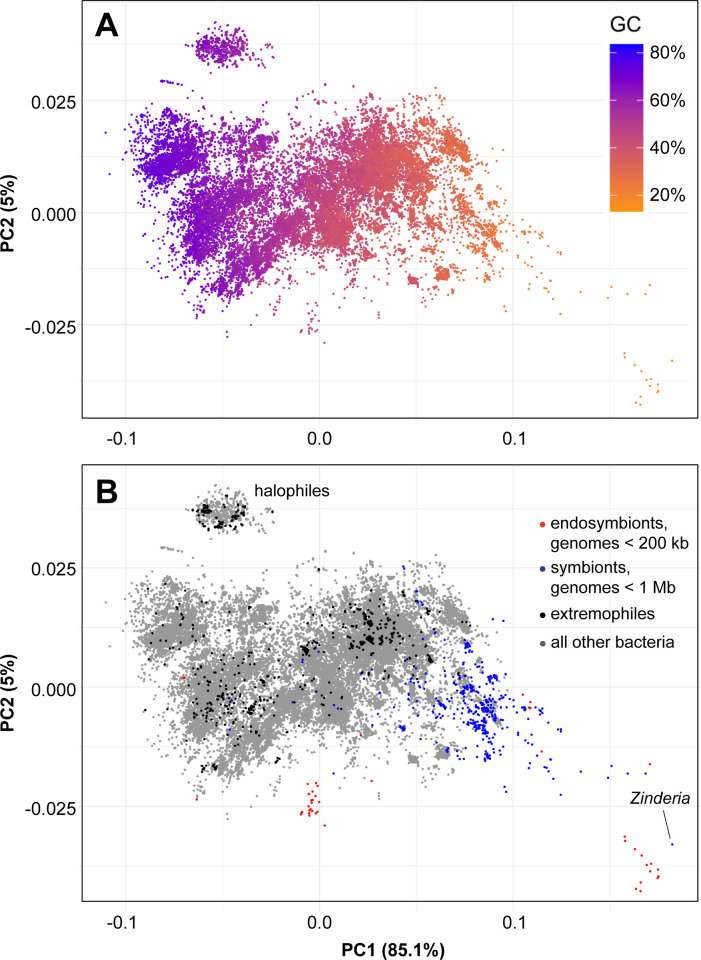
Endosymbionts proteins have extreme compositional biases. A principal component analysis (PCA) of amino acid frequencies from 98,966 bacterial and archaeal genomes. Each dot represents an amino acid profile from a single genome. Both A and B show the same data, where PC1 contains 85% of the variance and PC2 5%. **(A)** The GC content of the genome is shaded from blue (high GC content) to orange (low GC content), showing that the variation in amino acid frequencies are mostly driven by the GC content of the genome. **(B)** Representative extremophiles are colored black, organisms with genomes of less than 1 Mb in length are colored blue, organisms with genomes of less than 200 kb are colored red, and all other organisms are colored grey. Endosymbionts tend to be on the right side of this plot, reflecting their low genome GC content, and many endosymbionts—especially tipping-point endosymbionts—are well off the main cloud of prokaryotic proteome variance, likely reflecting their rapid rates of sequence evolution. *Zinderia* is colored blue because it just missed our somewhat arbitrary cutoff of 200 kb. PCA was done using factoextra. Amino acid frequencies and GC content were calculated on proteomes from the RefSeq database using custom Python scripts, which, along with data files used in this analysis, are available here: https://zenodo.org/records/10780716.

Given the pervasive AT compositional bias in endosymbiont genomes, and because endosymbionts are well established to have very high rates of sequence evolution [22,23], we wanted to visualize the amino acid compositions in endosymbionts relative to all other bacteria. In particular, we wanted to see how the amino acid composition of tipping-point endosymbiont proteomes compared to proteomes from other bacteria. We used principal component analysis (PCA) to display the amino acid compositions of approximately 100,000 bacterial and archaeal proteomes (Fig 3). We first confirmed that the primary driver of amino acid composition bias in prokaryotic proteins is the GC content of the genome (PC1, x-axis, accounting for 85% of the variance; Fig 3A). We note that the strong relationship between GC content and amino acid frequencies was recapitulated using only the frequencies of amino acids in a proteome as input to the PCA (no information about genome GC content was used in the analysis). We do not know what explains the amino acid variation in PC2 (y-axis), but, interestingly, some archaeal and bacterial halophiles (that is, organisms that grow in high salt conditions) are enriched at one end of PC2 (cloud of points at the top left of Fig 3B), and some bacterial endosymbionts on the other extreme (blue and red points at the bottom right of Fig 3B). We stress that the analyses shown in Fig 3 are preliminary and further work will be needed to better understand the patterns we report here.

While the GC content of an organism’s genome influences the distribution of amino acids in that organism’s proteins, selection for protein structure and function should impose limits on this distribution. We wondered if extremophiles, or organisms that live at the physical limits of life such as high or low temperature, pH, or salinity [24], might have proteomes that existed at the outer limits of the prokaryotic amino acid composition distribution. Aside from the halophiles, we find that most extremophile proteomes are contained within the main cloud of bacterial variance (black dots in Fig 3B). We note that the large number of sequences that can fold into the same structure makes finding simple relationships between proteome composition and growth environment difficult [25,26]. Despite this difficulty, some correlations between certain sets of amino acids and optimal growth temperature [27] and salt tolerance [28] have been found.

An intracellular bacterium resides in a place replete with nutrition and buffered from large swings in temperature, pH, and ionic concentration. But this stability is not reflected in amino acid compositions: Tipping-point endosymbionts have some of the most biased proteomes in bacteria (red dots in Fig 3B). This extreme amino acid bias suggests that in some circumstances, mutational and population genetic forces can be more powerful in driving outlier proteome compositional biases than physical forces such as salinity, temperature, and pH.

## How do you build a cell boundary with no genes to make it?

**Fig 4:**
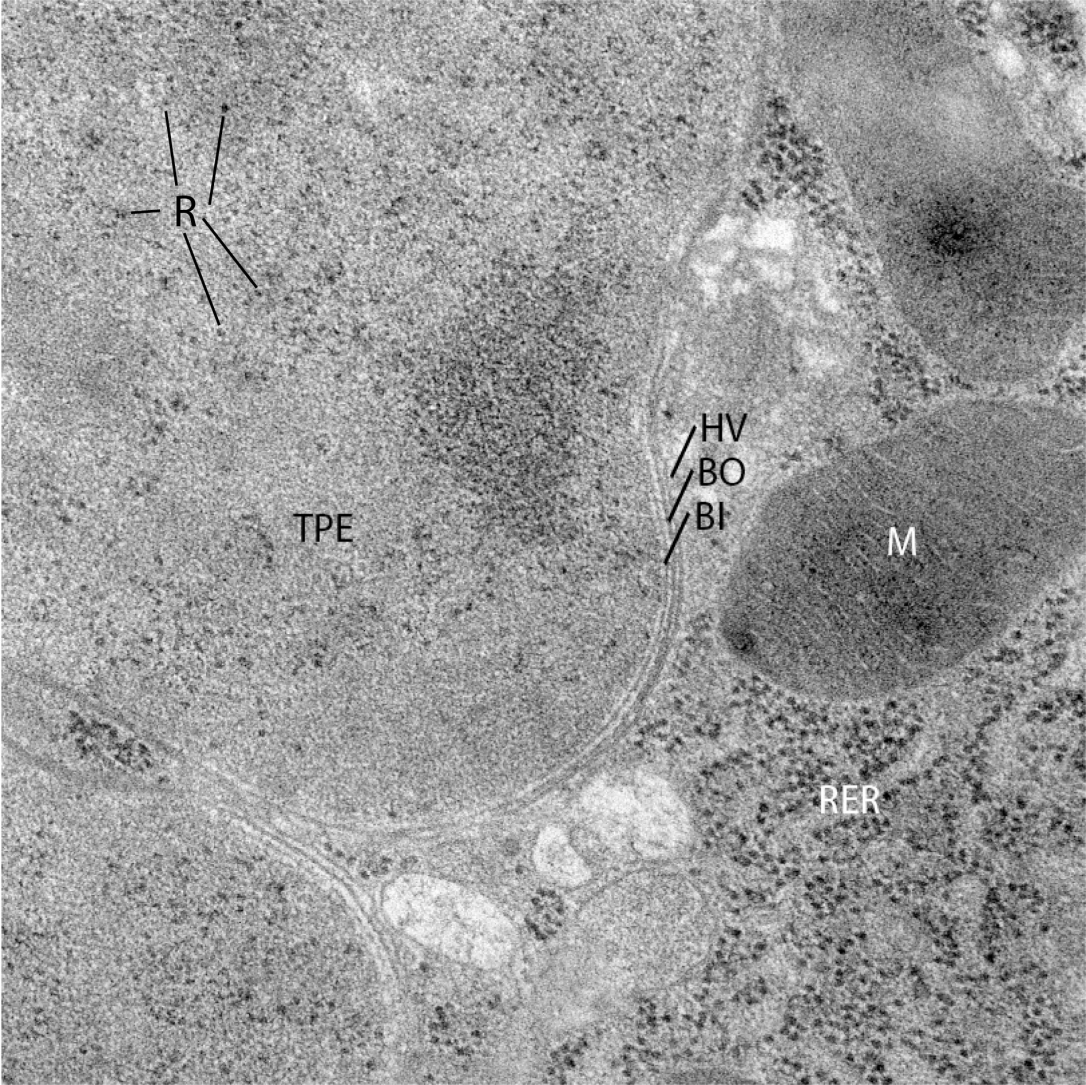
Cellular structure of an endosymbiont with a tiny genome. A transmission electron micrograph of a tipping-point endosymbiont (TPE) inside an insect cell. Image is of the endosymbiont *Tremblaya princeps* from the mealybug *Planococcus citri* and is courtesy of Dalton Leprich of Arizona State University. BI, bacterial inner membrane; BO, bacterial outer membrane; HV, host vacuolar membrane; M, insect mitochondrion; R, a few TPE ribosomes; RER, rough ER in the insect cytoplasm; TPE, the cytoplasm of the endosymbiont.

Like all cells, bacteria are defined by their envelopes, which separate their internal contents from their external environment. Ancient and conserved bacterial envelope features like peptidoglycan and lipopolysaccharides provide not just shape, structure, and stability but also unmistakable bacterial identity, enabling, for example, the cell’s recognition by eukaryotes as a pathogen [29,30] or as a prey item [31]. As we show in Fig 2, tipping-point endosymbionts have not only lost genes for producing these immune-stimulating molecules; they have lost all genes to produce any component of the bacterial cell envelope. Tipping-point endosymbiont genomes encode no genes for making their own cell membranes or for producing the lipid molecules from which membranes are assembled or the enzymatic machinery that enables transport of small molecules or proteins across membranes (Fig 2). But their membranes exist (Fig 4), and apparently work, so where do they come from?

It seems likely that, just as in mitochondria, chloroplasts, and plastids of secondary origin such as the apicoplast of apicomplexan parasites [32], tipping-point endosymbiont envelopes are provided by their host cell. In electron micrographs, many tipping-point endosymbionts show 3 lipid bilayers (Fig 4). The inner 2 membranes are presumed remnants from the ancestral diderm structure of their gram-negative ancestors, with the outermost third membrane added as a result of engulfment by their host cell [33]. Tipping-point endosymbionts are no longer elegant sphere- or rod-shaped cells like their free-living ancestors but are often large and irregularly shaped blobs [34,35]. How these 3-membrane blobs are constructed remains a mystery.

A useful comparison comes from intracellular pathogens, which, like host-beneficial endosymbionts, often have reduced sets of genes to produce cell envelopes [36] and reside in host vacuoles [37]. Pathogens maintain growth and structural support by scavenging eukaryotic membranes and membrane components from their hosts through redirection of existing intracellular transport pathways [38–41]. Pathogen exploitation of the host endomembrane system is typically mediated by the secretion of proteins called effectors into the host cell, a process that is likely important to the early evolution of host-beneficial endosymbioses [42] but may not explain tipping-point endosymbiont function because the autonomous ability to secrete effectors has been lost.

So what possible pathways remain for building tipping-point endosymbiont envelopes? Mitochondria and chloroplasts, in part, build their membranes at close junctions with the host endomembrane system called membrane contact sites, where lipids can be passed from closely spaced membranes through protein junctions [43–46]. These contact sites are also formed between many other organelles in the cell and, perhaps relevant to tipping-point endosymbionts, are thought to occur at the host–endosymbiont interfaces of secondary plastids [47,48]. Secondary plastids are formed when a whole single-celled photosynthetic eukaryote (containing a chloroplast) is taken up by another (nonphotosynthetic) single-celled eukaryote, giving the new secondary plastid not 2 but 4 surrounding membranes [49,50]: 2 membranes from the original plastid, 1 from the plasma membrane of the engulfed cell, and 1 from the additional plasma membrane of the engulfing cell added during phagocytosis. Similarly, the outer (third) membrane of many tipping-point endosymbionts is formed from phagocytosis by a eukaryotic cell of a bacterium with an ancestral diderm membrane structure [33] (for example, the membrane labeled HV in Fig 4). While little is known about how these outermost host membranes are recognized by the host cell (as ER-like, or as something more akin to a stalled endocytic vacuole, etc.), studying their connections to the rest of the host cell through structures like membrane contact sites may be a decent place to start to better understand the mechanisms of long-term endosymbiotic associations.

## How do you transport molecules across the cell envelope with no genes to make transporters?

Lipid bilayers are semi-impermeable barriers that allow only small, noncharged molecules such as water, CO_2_, and some small metabolites to move across them at biologically meaningful rates [51]. The transport of charged or larger molecules such as protons, ions, most amino acids, proteins, and RNAs must therefore be vesicle or transporter mediated. Most known tipping-point endosymbionts are nutritional endosymbionts whose primary role is to make essential amino acids and vitamins for the host cell. How are these products transported out of an endosymbiont that cannot make membrane proteins, and how are the building blocks for these essential compounds transported in?

The mechanisms for amino acid transport at the outermost host vacuolar membrane (HV in Fig 4) are understood in some sap-feeding insects. These insect hosts have evolved new amino acid transport proteins through gene duplication, which seem to function specifically in symbiosis [52], and the host enriches the endosymbiont-containing vacuolar membrane with amino acid transporters [53]. This still leaves 2 membranes, which must be crossed to transport nutrients between endosymbiont and host (BO and BI in Fig 4), because tipping-point endosymbionts lack transporters in (and the ability to autonomously insert transporters into) either of these membranes. How this metabolite transport happens through these inner 2 membranes is unknown.

More problematic still are what the massive losses in other fundamental bacterial pathways imply for RNA and protein import into tipping-point endosymbionts. Genomic data suggest that some endosymbionts require horizontally transferred bacterial genes present on the host genome, and presumably made in the host cytoplasm, to function [54–56]. If these proteins are targeted to the tipping-point endosymbiont cytoplasm, this must be a host-directed process. This would seem to require either specialized vesicles with targeting signals directing them to the endosymbiont or an endosymbiont-specific protein translocation machinery (possibly co-opted from mitochondrial protein import through the insertion of ancestrally mitochondrial translocases and use of transit peptides).

The evolution of such a protein targeting and import system seems like a huge leap, and it has often been described as a major milestone in the evolution of a bona fide organelle [57,58]. Landmark experiments surveying the protein content of small-genome (but pre-“tipping point”) endosymbionts in unicellular eukaryotes have shown that host protein import does indeed occur on a large scale [59,60]. There is also some evidence suggesting that sap-feeding insects, including the hosts of tipping-point endosymbionts, have the ability to import proteins and other large molecules into their endosymbionts. Using fluorescently tagged antibodies to visualize proteins within insect tissue, host-encoded proteins have been identified in the cytoplasm of aphid and mealybug endosymbionts [55,61]. These latter analyses focused on just one protein each, but, like the unicellular eukaryote examples above, the total number of imported proteins in each of these cases is likely to be much higher. The mechanisms of this import remain unknown.

## How do you make proteins when missing key translation-related genes?

All organisms use the same highly conserved set of enzymes to translate mRNA transcripts into proteins: a ribosome to ligate growing chains of amino acids, tRNAs to carry amino acids to the ribosome, and aaRSs to ligate amino acids to tRNAs. Likely due to the ancient and fundamental nature of this process, genes involved in translation are also some of the last to be lost from even the most reduced endosymbiont genomes [7,8]. All bacterial (and organellar) genomes retain ribosomal RNA (rRNA) and most retain a minimal set of tRNA genes capable of decoding codons for 20 amino acids (Fig 2). Because tipping-point endosymbionts still maintain ribosomes (Fig 4) that apparently make proteins [62], the loss of tRNAs or proteins essential for translation beyond this minimal set necessitates compensatory adaptations or functional replacements to maintain protein synthesis. The identities of these structural adaptations or the replacing molecules are unknown but have fascinating consequences for the way these bacteria now perform protein synthesis.

While most endosymbiont genomes have converged on a minimal set of tRNAs capable of decoding codons for all 20 amino acids, some tipping-point endosymbionts have lost numerous tRNA genes (Fig 2). For example, 1 cicada endosymbiont is missing tRNA genes to decode leucine, valine, arginine, serine, threonine, aspartic acid, asparagine, and tyrosine codons (but still has genes containing these codons) [63]. This loss is even more impressive when considering that almost all bilaterian animal mitochondrial genomes have the same core set of 22 tRNA genes, resisting almost 2 billion years of genomic erosion [64]. One barrier in tRNA replacement appears to be the lack of functional horizontal gene transfer of tRNAs: There is not a single reported case of a bacteria-to-nuclear tRNA gene transfer being imported by an organelle or endosymbiont [65]. Without the transfer of bacterial tRNA genes, the source of the replacing tRNAs in tipping-point endosymbionts would seem to be the host’s eukaryotic tRNAs. However, bacterial and eukaryotic tRNAs are divergent in sequence and make poor reciprocal substrates for aaRSs [66,67]. How, or if, these eukaryotic tRNAs become targeted to and imported by tipping-point endosymbionts, and how these molecules interact with the remaining bacterial translational machinery, is, again, unknown.

The faithful decoding of the genome requires the correct amino acid be ligated to the correct tRNA by the correct aaRS [68]. The tightly evolved relationship between aaRS and tRNA may be one reason why these enzymes are some of the last proteins to be lost from reduced bacterial genomes. But unlike tRNAs, aaRSs have been functionally transferred across all 3 domains of life [69]. Eukaryotic nuclear genomes typically encode 2 separate sets of aaRSs, a set for cytosolic translation and another for mitochondrial and chloroplast translation, with the organellar synthetases often being of bacterial origin [70,71]. This means that more recent bacterial endosymbionts find themselves in hosts that already encode multiple aaRS enzymes, some of which are of bacterial origin and already trafficked to other organelles of bacterial origin such as the mitochondrion and plastid. In cases where aaRSs are finally lost from tipping-point endosymbiont genomes, the answer to their replacement could involve multiple scenarios. One possibility is that new (not those already existing for organelle translation) bacterial aaRS genes are transferred to the host to be used by tipping-point endosymbionts, although genomic work to date has found no evidence of this occurring [54,56,63]. Other possibilities include the import of organellar or cytosolic aaRSs into tipping-point endosymbionts, or, bypassing aaRS import altogether, the import of charged tRNAs from the host [65,72].

And, finally, at the heart of translation is the ribosome, a large, protein-assembling complex composed of a catalytic core of RNA and a highly conserved set of supporting proteins. If a cellular genome exists, it encodes ribosomal RNA: rRNA is one of the only universally retained elements in all cellular genomes [73,74]. Although some ribosomal proteins have a variable presence in genome evolution (Fig 2), a complement of about 50 proteins is tightly conserved across bacteria [75]. The loss of multiple (up to 20 [76]) ribosomal proteins in some tipping-point endosymbionts raises 2 (not mutually exclusive) possibilities for how these ribosomes continue to function. The first possibility is that tipping-point endosymbiont ribosomes are being reduced to a minimal, but functional state. In this scenario, ribosomal protein losses are eroding away the accumulated outer shell of the modern ribosome to a macromolecular complex perhaps more similar to its ancient functional core [77,78]. The second possibility is that these ribosomal protein losses are being replaced by proteins imported by the host, similar to how mitochondrial ribosomes have accumulated new host-derived ribosomal proteins [79,80]. Either outcome is interesting and will require detailed structural work to solve. Overall, the massive loss of translation-related RNAs and proteins—the most highly conserved gene products in cellular evolution—is a striking feature of tipping-point endosymbionts and will be an important area of future research.

## How do proteins function with extreme amino acid compositional biases?

A considerable amount of evidence suggests that endosymbiont RNAs and proteins do not work particularly well. Endosymbiont rRNAs [81], tRNAs [82], and proteins [83] are all predicted to be less structurally stable than those from their free-living relatives. Endosymbiont cells are more heat sensitive than their insect host organisms [84,85], and this increased sensitivity is likely in part due to less thermostable proteins. Some endosymbiont proteins are promiscuous in their biochemistry, which may in part enable the functional loss resulting from genome reduction [86]. The editing and tRNA discrimination domains of aaRSs from intracellular bacteria are divergent in ways that likely affect the fidelity of translation [87]. Collectively, these data point to an overall impaired, or at least highly divergent, biochemistry in endosymbionts.

How do endosymbionts deal with these unusual proteins? One solution seems to include the high constitutive expression of protein chaperones, or enzymes that help misfolded proteins achieve their final, properly folded structures [88]. Specifically, high levels of the protein chaperone GroEL in endosymbionts is thought to buffer the effects of having proteins with less stable structures [22,89,90]. But while it has long been known that many different linear strings of amino acid sequences can fold into the same 3D structure, the amino acid biases in some tipping-point endosymbionts are far beyond what is seen in most other organisms. For example, *Zinderia* (Fig 3) has a proteome where almost 50% is just 3 amino acids (18% lysine, 17% isoleucine, and 13% asparagine [20]), whereas the averages for these amino acids in bacteria are 5%, 6%, and 4%. Understanding how organisms compensate for these highly skewed amino acid sequences would be of interest, whether it be by high chaperone expression or compensatory changes in 3D structure to, for example, accommodate large amounts of positively charged lysine residues, or both. Of particular interest would be understanding how these skews in amino acid compositions affect multiprotein complexes, or RNA–protein complexes such as the ribosome. Large amounts of positively charged amino acids are not tolerated in the hydrophobic core of proteins [91], and so these residues are almost certainly biased towards the exposed outer shell of the protein, where protein–protein and protein–RNA interactions take place. Solutions to these riddles will come from biochemical studies on isolated proteins and RNAs, but also, once again, from inspiration from mitochondrial and plastid biology, where scientists have long considered the complexities of rapid and uneven sequence evolution across dispersed (but interacting) symbiotic genomes and cellular compartments [74].

## Conclusions

Here, we have highlighted several unsolved mysteries related to how endosymbiont-host systems function. These are difficult problems to solve. It is one thing to purify a protein complex from *Escherichia coli*, or an organelle from *Saccharomyces cerevisiae*, it is quite another to do these same experiments in a tiny insect, without genetic control, where the endosymbiont of interest exists only in a small, specialized tissue. The issue, of course, is that it was only by the study of strange insects and difficult-to-isolate protists in the first place that allowed these organelle-adjacent endosymbionts to be discovered. So we have no choice, and it is not all bad news. If genomics was the gateway into tipping-point endosymbiont biology, advances in chemical biology (such as click-chemistry), structural biology (such as cryo-EM), and genetics (such as CRISPR and RNAi) are the tools that will make the cell biological and biochemical study of nonmodel endosymbiont-host systems accessible, if not easy. Creative uses of these and other technologies over the next several years will allow at least partial answers into how endosymbionts work with so few genes.
